# Overcoming language barriers, enhancing collaboration with interpreters – an interprofessional learning intervention (Interpret2Improve)

**DOI:** 10.1186/s12909-022-03213-0

**Published:** 2022-03-12

**Authors:** Franziska Krampe, Götz Fabry, Thorsten Langer

**Affiliations:** 1 Center for Pediatrics, Department of Neuropediatrics and Muscle Disorders, Medical Center - University of Freiburg, Freiburg, Germany; 2 Present address: Childrens Hospitals Harlaching and Schwabing, Klinikum Rechts der Isar, Technical University Munich, Ismaninger Straße 22, 81675 Munich, Germany; 3 Department of Medical Psychology and Medical Sociology, Albert-Ludwigs-University Freiburg, Rheinstraße 12, 79104 Freiburg i. Br, Germany

**Keywords:** Interprofessionalism, Language barriers, Cultural competence, Interpreters, Education, Paediatrics

## Abstract

**Background:**

Language barriers (LB) are common in patient care. They can negatively impact the quality of care, and increase costs. LB can be overcome by using interpreters. However, collaboration with interpreters is a professional activity which can and needs to be learnt. *Interpret2Improve* is an innovative educational intervention where medical and nursing students learn together how to address LB and effectively collaborate with interpreters.

**Methods:**

The three-hour course has two parts: After a short introduction on the relevance of LB and resulting issues of patient safety etc., students in interprofessional teams of two practice conversations with non-German-speaking simulated patients and professional interpreters. The course is evaluated in a pre-post format with the *Freiburg Questionnaire for Interprofessional Learning Evaluation* which has been validated in prior studies.

**Results:**

Fifty-one students (thirty of the participants were medical students, 21 participants were students in nursing care) participated from 11/2016–07/2018. Overall, the course was very well received (mean 1.73 (SD 0.85) on a five point scale: 1 = very good, 5 = insufficient). The evaluation by medical and nursing students differed significantly. Fourteen out of twenty-one items show a self-assessed increase in interprofessional knowledge or skills.

**Conclusions:**

Students felt that their skills in addressing LB by effectively collaborating with interpreters increased during this interprofessional format. Further studies are needed to obtain further evidence beyond self-assessment and regarding the long-term outcomes.

**Supplementary Information:**

The online version contains supplementary material available at 10.1186/s12909-022-03213-0.

## Background

Healthcare systems in many countries are faced with increased immigration which leads to a growing ethnic, cultural and linguistic diversity [1]. In 2018, 1 billion individuals have been on the move or have moved to another country worldwide. In the European Union, 36.9 million people living in an EU Member State were born outside of the EU-28 in 2017 [2]. In Germany, 23.4% of inhabitants are of migrant origin, i.e. at least one parent was born in another country. Among children under 15 years of age the proportion is 37,5% [3].

As a consequence of an increasing linguistic diversity, language barriers (LB) present an important challenge to many healthcare systems. LB can impair access to health care service for patients who are not proficient in the language of the societies’ majority [4]. LB have a negative impact on the quality of care [5–7] and patient safety [8, 9] and increase costs through more frequent unnecessary testing, avoidable hospitalizations and longer hospital stays [10–14]. Thus they contribute to health disparities in many societies [15].

LB can impair the quality of communication between patient and clinician in numerous occasions such as history taking, counselling or patient education [16].

In fact, even when interpreters are available at low or no costs, many clinicians decide not to collaborate with them because they are dissatisfied with their own skills in dealing with interpreters [17–19] or they often recognize language barriers late in the care process [20].

It is common to use informal interpreters like family members or hospital employees for interpreting. However, informal interpreters make more mistakes than professional interpreters which can lead to more treatment errors [6, 21]. Further, informal interpreters rarely take on a neutral role and exert a stronger influence on the course of the conversation [22, 23]. Finally, many clinicians rate themselves to be competent in working with interpreters while in fact they are not [24].

However, even with professional interpreters being present, communication in a triad consisting of patient, clinician and interpreter differs significantly from the dyadic structure in most language-concordant encounters [25, 26]. Despite the traditional view held by many clinicians and interpreters themselves [27], interpreters in the health care setting do not act merely as conduits or “language switching operators” transforming messages from one language to another [28]. In fact, linguistic research has shown that interpreters act as co-producers of the conversation. For example, Bolden demonstrated how interpreters “assist” clinicians in taking a history. Interpreters in her study not just interpreted back and forth between clinician and patient, but also asked additional questions to objectify symptoms and by conveying the information to the doctor in a medically framed way [29]. While interpreters take on a supporting role in many situations, the opposite has been reported, too. In an analysis of video-recordings of routine diabetes review consultations, Seale et al. showed that interpreters frequently did not translate patients’ utterances they deemed unrelated to the diabetes [30].

As complexity of a conversation increases when interpreters are present it seems important that clinicians are adequately prepared for such encounters. One way to address this issue is education of care providers. Several educational programs for physicians, medical students, physician assistant students and pharmacy students have shown to increase the likelihood to work with professional interpreters and to improve the quality of their collaboration [31–39]. However, opportunities for clinicians to develop such competencies are still scarce. A survey investigating the medical curricula in 12 European countries showed that abilities to work with interpreters are rarely included [40]. For nurses and clinical psychologists, the authors are unaware of any published studies on training programs. In Germany, the need for more educational offerings in the context of cultural competence and global health including the collaboration with interpreters has been acknowledged in a recent position paper [41].

To address this gap we developed an educational intervention bringing together final year medical students and 2nd and 3rd year students in nursing in an interprofessional learning experience. Interprofessional Education (IPE) is defined as learning together, from and about each other from members of two or more professional groups to activate effective collaboration and improve the care of patients [42]. We found an IPE approach particularly suited for this topic because conversations with interpreters are interprofessional per se. Moreover, several health professions deal with language barriers in differing contexts which offers an interesting learning opportunity as different perspectives can enrich the learning experience. The intervention aims at

- Increasing awareness of the role of LB with regard to the quality of medical and nursing care
- Teaching strategies how to recognize LBs
- Teaching strategies how to collaborate with interpreters more effectively in a simulated scenario
- Providing insights how LBs specifically influence the work of medical and nursing staff, respectively

In this paper, we describe the intervention and present the results of the evaluation study.

## Methods

The aim of this study was to evaluate the feasibility of the collaborative learning module and to evaluate its effect from the learners’ perspectives. The study was designed as a mixed methods study using qualitative and quantitative data in a pre/post evaluation (self-report).

### Theoretical and didactic framework

The course development was conducted using the Program to Enhance Relational and Communication Skills (PERCS) as a didactic framework. The PERCS pedagogy is based on the concepts of validating clinicians’ existing relational capacities, emphasizing moral dimensions of care, suspending hierarchy, and creating a safe learning environment [43, 44]. Another important element is the use of simulated patients (SP) portraying the roles of patients and family members. The SP have received special training to assist with debriefing and give participants direct feedback on their communication styles and approaches [45, 46]. We chose PERCS as a framework as it combines important elements of relational learning with the opportunity to facilitate interprofessional discourse in the context of interprofessional education. The learning objectives are displayed in Table 1.

**Table 1:** Learning objectives of the course

After the seminar the participants can ♣ explain the importance of language barriers for the quality of care in medicine and nursing care, ♣ work effectively with interpreters, ♣ explain the relevance of interprofessional cooperation and ♣ describe the professional roles of colleagues from other professions	

### Description of the intervention

The course is designed as a three-hour seminar. It is part of a longitudinal interprofessional curricular thread at the Faculty of Medicine Freiburg [47]. The data presented here were collected from winter term 2016/2017 to summer term 2018. Both medical and nursing students learn at the University Hospital in Freiburg, Germany. In Germany, medical training is a six-year-program. The participating medical students are all in the final year of training in which they are part of care teams and work under supervision of senior medical staff. The training as a nurse takes 3 years. The participants are in their 2nd and 3rd year of training in which practical assignments and theory units alternate. Both professions have had first experiences in patient contact at the time of the course. The interprofessional teaching team consists of a paediatrician with expertise on social paediatrics and language barriers, a medical psychologist with a focus on research on communication, the head of the local interpreter pool, as well the trainer for the simulated patients who also has a background in nursing. The language of instruction is German. Medical and nursing students in the appropriate year of training were sent an email asking whether they would like to take part in the course. Therefore, it was a convenience sample of participants. Participation was voluntary.

### Content and structure of the course (see Fig. 1)

#### Theoretical introduction

The participants learn about the relevance of language barriers in medicine and nursing in terms of quality of care and patient safety. Important topics include the frequency of language barriers, the diversity of languages spoken in Germany, the ethics of language barriers regarding equal treatment, legal aspects regarding the validity of informed consent conversations.

**Fig. 1:**
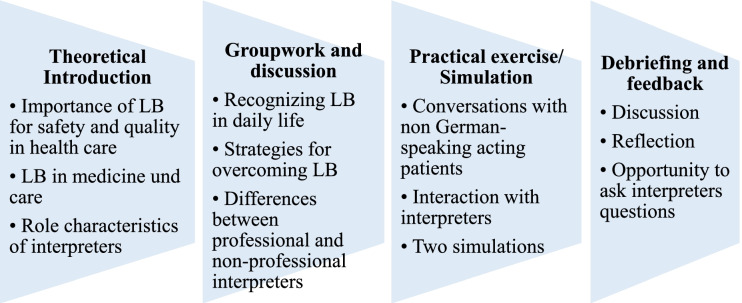
Content and Structure of the course (LB = language barriers)

#### Groupwork and discussion

In a subsequent exercise, participants are invited to share their own experiences in the context of the topic and to develop ideas on how to recognize and overcome language barriers in everyday clinical practice. The results are visualized and serve as a starting point for the following discussion focusing on differences and similarities among participants’ experiences. During this group discussion, the participants also learn about different variants of interpreting (telephone interpreting, professional vs. non-professional interpreters) and the respective advantages and disadvantages associated with the different options and the potentially resulting problems.

#### Practical exercise / simulation

Next, participants have the opportunity to practice an enacted, interpreted conversation with a SP speaking in a foreign language and the linguistically appropriate interpreters. SP cases include informing a mother about the HPV vaccination of her daughter or taking the history of a toddler with gastroenteritis and a diaper rash who is presented in the emergency department by his grandmother (see Additional file 1). The SPs are trained to portray these cases authentically. We offered the case studies in Russian, Turkish, Spanish and Portuguese. Thus, participants experience the similarities and differences between an interpreter-mediated and a language-concordant conversation. We offered two simulations per teaching session in which we used different cases. The simulation interviews can either be done by a participant alone or as an interprofessional team consisting of a medical student and a student of paediatric nursing. The participants are instructed to focus not only on the medical issue at hand (e.g. differential diagnoses of gastroenteritis or data regarding the incidence of HPV-associated diseases) but are also encouraged to address the psychosocial cues offered by the SPs.

#### Debriefing and feedback

After the simulations, participants of the role-play engage in a debriefing exercise with the SPs, the group and course leaders [48]. On the one hand, this discussion serves as feedback for participants regarding their performance. Therefore, interpreters gave the participants structured feedback following the enactments.

On the other hand, special features emerging during the simulation are being reflected in the group for a more general discussion. Typical topics include the introduction of interpreters in the conversation with patients, the seating arrangement in interpreter-supported conversation or the use of short phrases during the conversation. After participating in or witnessing the simulation, participants are also able to ask interpreters questions e.g. regarding their interpretation of role-neutrality. In this way, participants engage in an interprofessional, reflective discussion about behavioural and context factors which should be considered by medical and nursing staff in order to optimize the quality of the interaction with interpreters and patients.

##### Evaluation

The intervention was evaluated by participants in a pre-post design using both online and paper/pencil questionnaires. Questionnaires were based on previously published PERCS surveys [44] and the Freiburg Questionnaire for Interprofessional Learning Evaluation (“FILE”) [49] and included questions on demographic characteristics. The items from exploratory PERCS surveys focus on the learner’s experience of the workshop (5 closed and 5 open questions). The FILE is an instrument for the self-assessment of different aspects of interprofessional competencies which has been psychometrically tested. It comprises 21 items and includes the following scales: *relevance of interprofessionalism* (10 items; CR-α = 0.90), *understanding of one’s role (5 items;* CR-α = 0.78*), abilit*y *to work in a team* (6 items; CR-α = 0.69), *team competence* (5 items; CR-α = 0.69). The items are rated on a five-point Likert scale (1 = very good, 5 = insufficient) [49].

##### Analysis

The evaluation was carried out by means of descriptive statistics (absolute and relative frequencies, group comparisons by T-test, Bonferroni correction) as well as an orienting qualitative content analysis of the free text answers [50]. For the statistical analysis we used SPSS (version 25.0 and 27.0, Armonk, NY: IBM Corp.).

## Results

### Demographic data of the participants

Fifty-one students participated in the course. Forty-three (84%) of the participants were female. Thirty (59%) of the participants were medical students, 21 participants (41%) were students in nursing care (Table 2).

**Table 2:** Description of the sample

	Medical students	Nursing care students
Female	22	21
Male	8	0
Age	22–36 years (M = 26.84 years)	20–48 years (M = 23.4 years)

On average the participants were 25.5 years of age (SD **±** 4.7 years)

### Overall assessment of the course

The course was rated with 1.73 (SD **±** 0.85) overall. The ratings improved after year 1 (winter semester 2016/17: m = 2.60 (SD **±** 1.27), summer semester 2017: m = 1.4 (SD **±** 0.5; summer semester 2018 m = 1.69 (SD **±** 0.6 (*p* < 0.01).

**Fig. 2:**
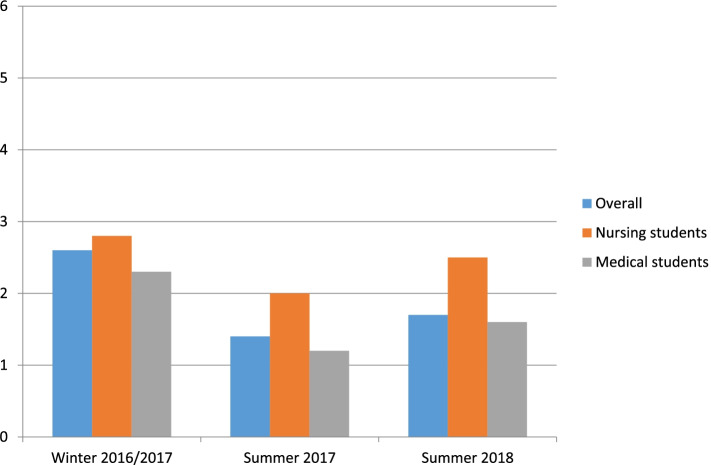
Evaluation (School grades 1–6, 1 = very good, 6 = insufficient)

Medical students rated the course better than nursing students (medical students: 1.5, nursing students: 2.1, *p* = 0.02). Some nursing students stated in the open-ended questions that they rarely take a history in their training and thus felt overwhelmed in the simulated conversations (see also Fig. 2).

Ninety Eight percent of the participants were in favour of a continuation of the course. This was supported by participants’ free text comments, e.g. *“continue this training, it is a very important topic which is increasingly needed in clinics”*. A paediatric nursing care students quoted *“I think it would be good if there were more interprofessional learning opportunities”*.

The teachers were rated at 1.43 (SD **±** 0.816), *n* = 49. No significant differences were found in the assessment of teachers between the individual professions.

### Evaluation of the interprofessional learning experience

**Fig. 3:**
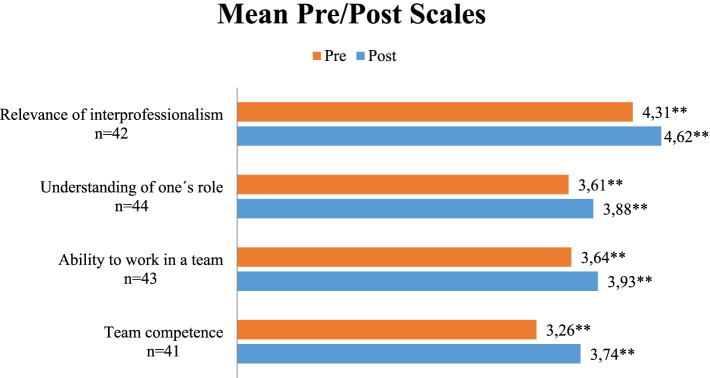
Mean Pre/Post of scale

In the pre/post comparison, participants showed an increase in all four scales covered by the FILE (*p* < 0.002 (see Fig. 3).

When looking at single items, 14 out of 21 FILE items show significant results regarding higher self-assessed competence after the course. The participants rated the item “The teachers were good representatives of interprofessional cooperation” (rated on a five-point Likert scale from “1 = does not apply at all” to 5 = “fully agrees”) after the course m = 4.60, *n* = 45 (SD ± 0.75). The item “My interest in interprofessional learning has grown through the event” was rated post as m = 4.50 (SD ± 0.61) with no pre item regarding this question. The item “Through the event, my role in the interprofessional team became clearer” was rated post as m = 4.08 (SD ± 0.86). These findings are supported by the answers in the open-ended questions. Participants stated that *“working together interdisciplinary for the patient!”* is important and they liked how the course *“brings together different professional groups”*.

### Evaluation of the learning experience with regard to language barriers and interpreters

Medical students said after the course they learned that *“Interpreters translate everything you say and what everyone in the room says. They stay strictly in the background”*. Another student stated: *“How to conduct the interview: seating arrangement, short sentences, that a preliminary conversation with the interpreters is important, explain to the relative what happens, seek consent; declare confidentiality to the patient”* as well as to have *“eye contact with patient – not with the interpreter”* were two of the main learning issues for medical and paediatric nursing care students. Several participants stated the want to **“***demand interpreters actively for better treatment quality”* in the future.

## Discussion

We found that bringing together medical and nursing student to learn about the relevance of language barriers and to improve collaboration with interpreters is both feasible and valued by participants.

The course was very well received by the participants with an overall grade of 1.73, with a significant difference in the rating of medical students compared to students in (child) nursing. This difference can be explained by the fact that the case studies used during the role-plays in the first two seminars were not sufficiently adjusted to the nursing students’ level of experience. Subsequently, we revised the gastroenteritis case and expanded it by specific topics of care such as hygiene and wound care. In the following seminars, an increase of the satisfaction with the case studies by the nursing students and because of that of the evaluation of the course in general between the winter term 2016/17 and the following semesters could be observed.

Regarding the learning objectives, participants stated that they learned about the importance of language barriers for the quality of care and how to work effectively with interpreters during the course. This is in line with evidence from other studies, showing that training for medical staff, in which participants learned about the importance of language barriers and practiced with interpreters, increases the willingness to work with professional interpreters [36, 51] as well as physicians’ respective competences [37]. Jacobs et al. also report this result from a one-and-a-half-hour training course with a similar curriculum for medical students [32].

Our intervention aimed at framing interpreter-supported conversations as an interprofessional activity. This was reflected by the teaching team which included health care professionals from different backgrounds and a trained interpreter. The positive evaluation of the course and the teaching team seem to acknowledge this setting. The relevance of interprofessionalism in healthcare was also highly valued with a significant increase in the before and after evaluation. After the course, the participants said that they critically reflected their actions in the interprofessional team in order to understand their own roles better and they also stated that learning together had a positive impact on their opinion about other health professions. This is in line with findings from previous studies suggesting that participants in an interprofessional course gained a clearer understanding of their role, critically reflected their actions in the interprofessional team, worked well with other professional groups and learned about their personal limits [52–57]. The scale of the “relevance of interprofessionalism” also showed a significant increase after the course. This is in line with previous studies in the interprofessional field, which have already shown that the relevance of interprofessionalism can be learned in a course [57]. This is important because the implementation of interprofessional teaching courses for students has so far been inadequate [58, 59].

Although, IPE has gained increasing attention among educators in many countries including Germany its implementation of learning opportunities can be challenging for conceptual and organizational reasons [56, 57, 60–74]. The course presented here requires resources for the development of cases and the training of SPs. However, it requires a relatively short teaching time and little teaching material. Nevertheless, recruiting participants was partly challenging due to number of reasons: The optional nature of the training and many compulsory lessons in students’ curricula; a presumed lack of reflection on the importance of the subject and the need to be trained in interpreting which is in line with prior studies indicating similar challenges regarding the organisation of such courses [57, 64]. On the other hand, frequent reminders of the students via different information channels (email, personal conversations, recommendations from participants from previous courses, the university’s course platform and flyers), as well as an early announcement and close consultation with the training managers were helpful for the organization. We expect that due to the increasing proportion of migrants in our society and the frequent encounter with language barriers in everyday clinical practice, the general willingness to participate in such courses will increase and clinicians will become increasingly aware of the need for such training. Offers such as video interpreting in the outpatient clinic or German courses for parents of chronically ill or long-term hospitalized children, might also contribute to bring the relevance of the topic to everybody’s mind [75].

Limitations of the present study are the relatively small number of participants with *n* = 51 as well as participation in the course on a voluntary basis. Thus, the results might be biased by prior interest in the subject and other motivational factors. An implementation of this interprofessional course as part of the compulsory curricula for students, as for example in Sweden [56, 60–62], would therefore be desirable but requires appropriate ressources. Furthermore, the reported learning success is based on the participants’ self-assessment rather than objective assessment. This should be addressed in follow-up studies.

The present interprofessional course at the Faculty of Medicine Freiburg [47], is, to our knowledge, unique in Germany. Since 2018, the seminar has been offered in a modified form for practitioners from medical and non-medical fields [76].

## Conclusion

Students felt that their skills in adressing LB by effectively collaborating with interpreters increased during this interprofessional format. The handling of LB and the effective collaboration with interpreters seems very well suited for an interprofessional course for medical and nursing students as well as other health professionals. With optional participation and a high density of other (compulsory) teaching lessons, recruiting participants is a challenge. Compulsory training courses for medical professionals dealing with language barriers and sensitization in the use of interpreters have been published, for example, in the US [31, 33–35, 38] and would be desirable in Germany as well. The extent to which the course contributes to a change in care practice should be further explored.

## Supplementary Information

**Additional file 1.**

## Data Availability

The datasets used and/or analysed during the current study are available from the corresponding author on reasonable request.
