# Supplementary material for: Overcoming language barriers, enhancing collaboration with interpreters – an interprofessional learning intervention (Interpret2Improve)
Source: BMC Med Educ. 2022 Mar 12;22:170. doi: 10.1186/s12909-022-03213-0 (PMC8918305; doi:10.1186/s12909-022-03213-0)
Supplement: Supplementary file 1 — Additional file 1. [file 12909_2022_3213_MOESM1_ESM.docx]

**Additional file 1: Supplementary information**

| Appendix A: Case study 1  Background: Ms. Gomez is 48 years old and from Madrid. She has been living in Freiburg for 8 months. She came to Germany with her husband and two children (11 and 7 years) as part of a research stay for two years.  Characteristics of the mother: Attentive, questioning and interested in conversation. She wants to make the right decision for her daughter.  External appearance: well-groomed appearance  Taking the history of the patient: Ms. Gomez has arranged a consultation to discuss the prsos and cons of having an HPV vaccine for her older daughter Paula. She wants to have the first conversation without the daughter, as she finds it too early to discuss cervical cancer and sexual intercourse with her daughter. Instead, she wants to first be clear about whether she should get the vaccine.  Behavior in conversation: Mrs. Gomez tries to be polite. She does not want to understand her need for information as distrust. Although she is otherwise a self-confident and analytically thinking woman, in the current situation in the consultation, she is a little uncertain. At first, it is unpleasant for her not to speak enough German; she speaks a few words of German.  Task/situation description for the lessons: As a medical student, you are currently completing a rotation in the outpatient clinic during the practical year. You conduct the consultation and refer for further questions, to the specialist, which comes later. As part of a pilot project, a Spanish-speaking Spanish interpreter works at the clinic, who can provide flexible and long-term planning. The registration informs you that Mrs. Gomez does not speak German, her mother tongue is Spanish and the interpreter is currently available. |
| --- |

Case study HPV vaccine

| Appendix B: Case study 2  Background: Viktor is 2.5 years old and the second child of the Ivanov family. The family of three, originally from Siberia, has been living in Germany for 8 years. One year ago, the grandmother of Viktor (the mother of the child’s father) moved to Germany and lives in a neighboring apartment. Currently, Viktor’s parents have gone to Regensburg for a wedding and Viktor’s grandmother takes care of him. So far she hardly had any opportunity to learn German.  Taking history and physical examination: Viktor has barely been drinking for three days. He vomits 2–3 times/day and has watery diarrhea (4–5/d). The skin around the anus and in the groin is very red and occasionally bloody. Viktor screams as soon as he evacuate his bowels. Ms. Ivanova has so far often changed the nappies, thoroughly cleansed the skin with soap and then creamed with a moisturizing lotion.  Characteristics of Ms Ivanova: Uncertain but determined in the care of her grandchild.  Behavior in conversation: Ms. Ivanova comes to the hospital because she is very worried about her grandchild. He has never had diarrhea and vomiting, and he does not know how to drink. She is afraid he might die if nothing is done. She knows she needs help. At the same time, she never has been alone in a German hospital. It makes her uncomfortable that she hardly speaks German. Maybe there is a doctor or a nurse who speaks her language?  Task for nursing student / situation description: As part of your training you are employed in the outpatient clinic of the Children’s Hospital. After examining the child by the doctor, you should advise Mrs. Ivanova on skin care in the nappy area. You primarily inquire how Ms. Ivanova has done the care so far and give her targeted recommendations. Within the framework of a pilot project, a Russian interpreter works in the clinic, which you can use flexibly and without any preliminary planning. |
| --- |

Case study gastroenteritis
